# Combined Effects of Ozone and Drought on the Physiology and Membrane Lipids of Two Cowpea (*Vigna unguiculata* (L.) Walp) Cultivars

**DOI:** 10.3390/plants6010014

**Published:** 2017-03-03

**Authors:** Deborah Moura Rebouças, Yuri Maia De Sousa, Matthieu Bagard, Jose Helio Costa, Yves Jolivet, Dirce Fernandes De Melo, Anne Repellin

**Affiliations:** 1 Institut d’Ecologie et des Sciences de l’Environnement de Paris, Faculté des Sciences et Technologie, Université Paris-Est Créteil, 61 Avenue du Général De Gaulle, 94010 Créteil, France; debmoura85@yahoo.com.br (D.M.R.); repellin@u-pec.fr (A.R.); 2 Departamento de Bioquímica e Biologia Molecular, Universidade Federal do Ceará, Fortaleza, P.O. Box 6029, 60455-760 Fortaleza, Ceará, Brazil; yurimaia@gmail.com (Y.M.D.S.); costajhe@yahoo.com.br (J.H.C.); fernandesdemelod@gmail.com (D.F.D.M.); 3 Unité Mixte de Recherche Ecologie et Ecophysiologie Forestières, Université de Lorraine, BP239, F-54506 Vandœuvre-lès-Nancy, France; yves.jolivet@univ-lorraine.fr; 4 Institut National de la Recherche Agronomique, Unité Mixte de Recherche Ecologie et Ecophysiologie Forestières, BP239, F-54280 Champenoux, France

**Keywords:** drought, ozone, stress combination, membrane lipids, cowpea

## Abstract

The interactive effects of drought and ozone on the physiology and leaf membrane lipid content, composition and metabolism of cowpea (*Vigna unguiculata* (L.) Walp.) were investigated in two cultivars (EPACE-1 and IT83-D) grown under controlled conditions. The drought treatment (three-week water deprivation) did not cause leaf injury but restricted growth through stomatal closure. In contrast, the short-term ozone treatment (130 ppb 12 h daily during 14 day) had a limited impact at the whole-plant level but caused leaf injury, hydrogen peroxide accumulation and galactolipid degradation. These effects were stronger in the IT83-D cultivar, which also showed specific ozone responses such as a higher digalactosyl-diacylglycerol (DGDG):monogalactosyl-diacylglycerol (MGDG) ratio and the coordinated up-regulation of DGDG synthase (*VuDGD2*) and ω-3 fatty acid desaturase 8 (*VuFAD8*) genes, suggesting that membrane remodeling occurred under ozone stress in the sensitive cultivar. When stresses were combined, ozone did not modify the stomatal response to drought and the observed effects on whole-plant physiology were essentially the same as when drought was applied alone. Conversely, the drought-induced stomatal closure appeared to alleviate ozone effects through the reduction of ozone uptake.

## 1. Introduction

Plants, as sessile organisms, are permanently subjected to changing environmental conditions that might compromise homeostasis, growth and even survival. As a consequence, plants have developed elaborate mechanisms to sense and respond to changes in their environment through appropriate adjustments. Water availability constitutes a major limiting factor for plant productivity and drought is considered one of the most influent abiotic constraint to crop yield [1]. Rising temperatures and altered precipitation patterns are two main components of climate change and both contribute to increase the frequency and intensity of drought episodes regionally. Air pollution is another aspect of the anthropogenic alteration of the environment. Among air pollutants, tropospheric ozone is considered the most detrimental to plants [2]. Ozone is a secondary product of photochemical reactions that involve nitrogen oxides and volatile organic compounds. Due to the increased emissions of these precursors from vehicle and industrial sources, tropospheric ozone has increased since the 1950s [3]. Although emission reduction policies have successfully limited the frequency and extent of high ozone episodes and despite recent evidence that tropospheric ozone has leveled off in the last decade, at least in the Northern hemisphere [4], background concentrations have now reached levels potentially damaging to crop production in several regions of the world [5].

As high temperature and irradiance promote both ozone production and water deficit, the occurrence of high ozone exposure during drought episodes is common in semi-arid regions. It is clear that the response of plants to a combination of stress conditions is unique and cannot be predicted from the response to the stresses applied individually [6]. The interacting effects of ozone and drought are still unclear as contradictory findings are reported in the literature [7]. Meta-analyses of the effects of ozone on trees [8] and crops [9] showed that drought tended to mitigate the adverse effects of the pollutant. It is assumed that the drought-induced stomatal closure limits ozone uptake, thereby protecting leaf tissues from the oxidative stress caused by the pollutant. Reciprocal cross-protection was observed in *Medicago truncatula* [10] but ozone can also in some cases exacerbate the effects of water stress [11]. For instance, in oak seedlings, ozone combined with drought caused a stronger malondialdehyde accumulation and larger biomass losses than drought applied alone [12]. Ozone can also cause stomatal sluggishness [13]: recent results suggest that ozone can disrupt the ABA-induced signal transduction pathway for stomatal control, which could in turn impair the ability of plants to respond to water stress [14]. Overall, the interactions between ozone and drought depend largely on the temporal distribution of ozone and drought episodes [7].

At the cellular level, drought promotes the production of reactive oxygen species (ROS), essentially in the chloroplasts where the drought-induced over-reduction of the electron transport chain can lead to the formation of singlet oxygen (^1^O_2_) and superoxide (O_2_^−•^) [15]. Ozone is itself a strong oxidant and its decomposition in the apoplast generates a range of ROS. In all cases, if the oxidative load exceeds the apoplastic antioxidant capacity, ROS can spread within the cell and add to the ROS produced endogenously [16]. ROS, being reactive molecules, can oxidize all types of cellular components [15]. In addition, ROS play a role in signaling and induce defense responses [17]. Potential interacting effects between drought and ozone may thus arise from the stimulation of ROS production as a common feature of the two environmental stresses. 

Membranes are among the first cellular components to perceive drought stress and the role of phospholipases and lipid-derived messengers in triggering signaling cascades has been largely demonstrated [18]. Likewise, the ozone-triggered intracellular signal transduction is initiated at the plasma membrane through mechanisms that involve lipid peroxidation and subsequent formation of signal molecules such as jasmonates, although the primary site of ozone reaction within leaf tissues is the extracellular matrix [19]. Besides their role in cell signaling, membrane lipids may also contribute to stress tolerance through the adjusting of membrane fluidity and the reorganization of cell components [20]. From this perspective, the effects of drought and ozone on leaf membrane lipids share many similarities. Both stresses induce a decrease in membrane lipid content [21,22], an inhibition of lipid biosynthesis [23,24], a stimulation of lipolytic activities [25,26] and a decrease in linolenic acid (18:3) content [27,28]. It appears that changes in membrane lipid metabolism play an essential part in plant responses to the combination of ozone and drought stresses.

In the present work, we examined the effects of drought, ozone and the combination of these stresses on cowpea (*Vigna unguiculata* (L.) Walp.). Cowpea is a staple legume crop in semi-arid regions of the tropics and subtropics, where meteorological conditions promote the co-occurrence of drought and ozone stresses. While the impact of water stress on cowpea has been extensively studied [21,29,30,31], the response of this crop to ozone is less documented [32]. In recent studies, the responses of cowpea to ozone have been investigated in Asian and African cultivars with respect to biomass production, yield, photosynthesis, nitrogen fixation and ROS detoxification [33,34,35,36,37]. Here, we investigated the response of a Southamerican (EPACE-1) and an Asian cultivar (IT83-D) to the combination of ozone and drought stresses. In addition to the characterization of the physiological responses of the two cultivars to ozone and drought, a specific attention was drawn to plastidial membrane lipid content and fatty acid composition as well as to the expression of genes encoding enzymes involved in membrane lipid biosynthesis and degradation. The results show that ozone and drought stresses induced contrasting responses in cowpea plants and bring new insights into the interplay between general and specific multi-stress responses.

## 2. Results

### 2.1. Physiological Responses of Cowpea Plants to Ozone and Drought Stresses

**Figure 1:**
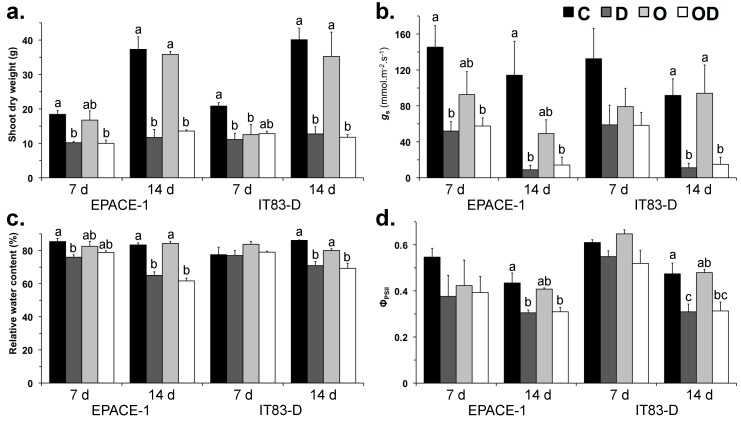
(**a**) Shoot dry weight; (**b**) stomatal conductance (*g*_s_); (**c**) relative water content and (**d**) quantum yield of PSII (Φ_PSII_) of *Vigna unguiculata* plants subjected to the following treatments: Control (C, black bars), Drought (D, dark grey bars), Ozone (O, light grey bars), Ozone + Drought (OD, white bars). Measurements were performed 7 and 14 days after the onset of the stress treatments. Means ± SEM are shown (*n* = 3–4). When significant differences were found, letters indicate homogenous subsets at each time point for a given cultivar (one-way ANOVA and Tukey post-hoc tests, α = 0.05).

**Figure 2:**
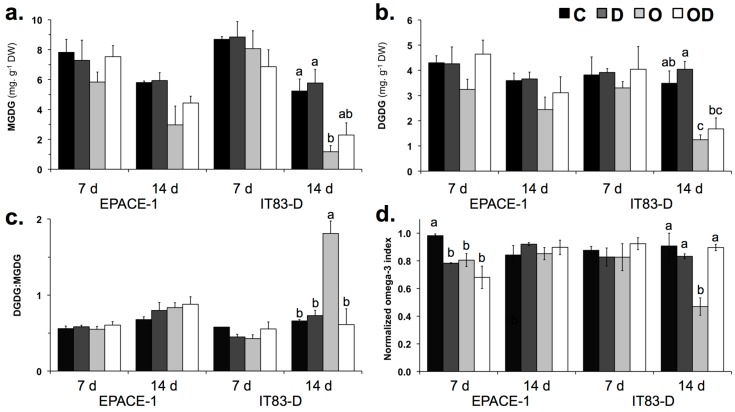
(**a**) MGDG contents; (**b**) DGDG contents; (**c**) DGDG:MGDG ratio and (**d**) normalized omega-3 index in the leaves of *Vigna unguiculata* plants subjected to the following treatments: Control (C, black bars), Drought (D, dark grey bars), Ozone (O, light grey bars), Ozone + Drought (OD, white bars). Measurements were performed 7 and 14 days after the onset of the stress treatments. Means ± SEM are shown (*n* = 3). When significant differences were found, letters indicate homogenous subsets at each time point for a given cultivar (one-way ANOVA and Tukey post-hoc tests, α = 0.05). MGDG, Monogalactosyl-diacylglycerol; DGDG, digalactosyl-diacylglycerol, omega-3 index 18:3/(18:0 + 18:1 + 18:2).

**Figure A1:**
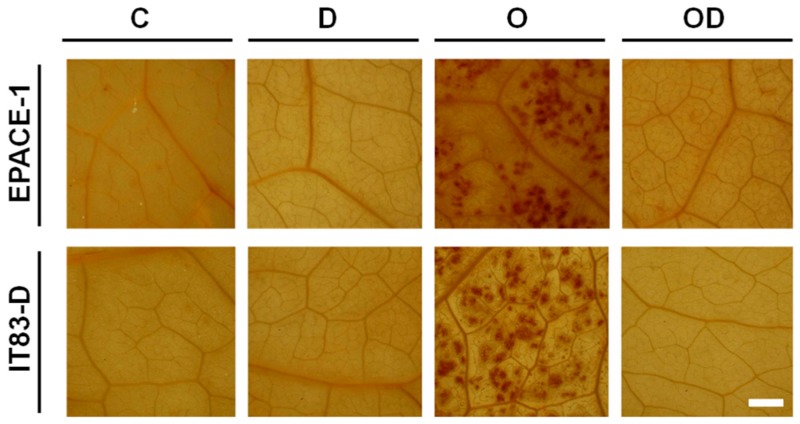
Detection of hydrogen peroxide using DAB staining in *Vigna unguiculata* leaves exposed to the following treatments: Control (C), Drought (D), Ozone (O), Ozone + Drought (OD). Leaf discs were collected and stained after 14 days of treatment. Pictures are representative of three independent biological replicates. No specific staining was observed in control leaves treated with ascorbic acid (data not shown). DAB: 3,3′-diaminobenzidine. Bar = 250 μm.

The individual and interactive effects of ozone and drought stresses on the two cowpea cultivars were first assessed at the whole-plant level on aerial parts by evaluating shoot biomass production (shoot dry weight, SDW), leaf water content (relative water content, RWC), stomatal conductance (*g*_s_) and chlorophyll fluorescence (effective quantum yield of photosystem II (Φ_PSII_) (Figure 1). Damage at the leaf level were evaluated by monitoring visible symptoms of damage, by detecting hydrogen peroxide (H_2_O_2_) (Figure A1) and by measuring plastidial membrane lipid contents (Figure 2). From now on, treatments will be referred to as C for control, O for ozone, D for drought and OD for the combination of ozone and drought. During the 14 d of experiment, plants of the O and OD treatments were exposed to an average of 130.4 ppb of ozone 12 h daily (Table 1). At the end of the experiment, the concentration-based index of ozone exposure AOT40 reached 16.7 ppm·h on average. The stomatal uptake of ozone (or Phytotoxic Ozone Dose, POD_0_) was 3.8 and 4.6 mmol·m^−2^ in EPACE-1 and IT83-D, respectively. Visible symptoms of leaf injury (dark/brown necrosis) and H_2_O_2_ accumulation were observed in both cultivars in response to the O treatment, to a greater extent in IT83-D than in EPACE-1 (Figure A1). However, no significant effect on biomass production, leaf water content, stomatal conductance or chlorophyll fluorescence could be observed in response to the O treatment, except a 40% decrease in shoot dry weight in IT83-D as compared to the control after 7 day (Figure 1a).

In contrast to the O treatment, the drought treatment alone (D) did not cause leaf injury or H_2_O_2_ accumulation (Figure A1) but had strong effects at the whole-plant level. Most of these effects were of the same degree in the two cowpea cultivars. First, the drought treatment induced stomatal closure as compared to the control, already after 7 day in EPACE-1 (−50%) and to a larger degree after 14 day in both cultivars (−90%) (Figure 1b). This response limited water loss as RWC was only 20% lower than in control plants after 14 day of drought stress (Figure 1c), but correlated with a severe restriction of plant growth: the aerial biomass of the drought-treated plants was 50% and 70% lower than that of the control plants after 7 and 14 day of experiment, respectively (Figure 1a). This strong restriction of above-ground biomass production was associated to a 35% decrease in the quantum yield of PSII (Φ_PSII_) in drought-stressed plants as compared to control plants for both cultivars (Figure 1d).

When the plants were exposed to drought in combination with ozone, the POD_0_ was 24% (2.9 mmol·m^−2^) and 33% (3.1 mmol·m^−2^) lower in EPACE-1 and IT83-D, respectively, as compared to plants treated with ozone only (Table 1). Consistently, the extent of leaf injury was less important in OD-treated than in O-treated plants and no H_2_O_2_ could be detected in leaves of the in OD-treated plants (Figure A1). At the whole-plant level, the effects of the combination of ozone and drought stresses were essentially the same as those of the drought treatment alone (Figure 1).

### 2.2. Galactolipid Content and Fatty Acid Composition

**Figure 3:**
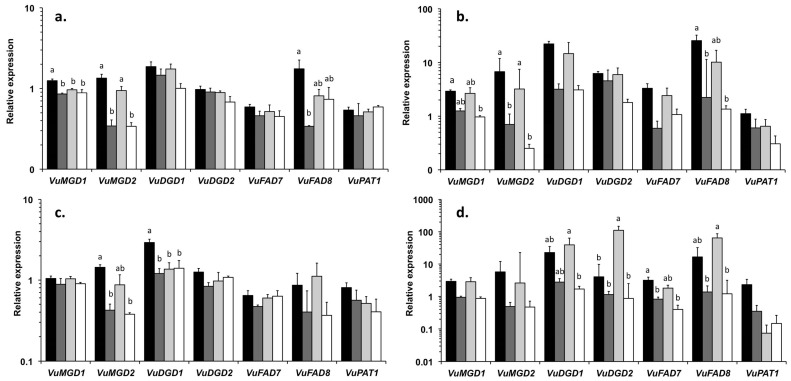
Relative expression of genes involved in lipid metabolism in leaves of (**a**,**b**) EPACE-1 and (**c**,**d**) IT83-D plants exposed to the following treatments: Control (C, black bars), Drought (D, dark grey bars), Ozone (O, light grey bars), Ozone + Drought (OD, white bars). Measurements were performed 7 and 14 days after the onset of the stress treatments. Means ± SEM are shown (*n* = 3). Each value is the average of two technical replicates. Expression of target genes was normalized to the expression of the reference gene *VuEF-1α*. When significant differences were found, letters indicate homogenous subsets at each time point for a given cultivar (one-way ANOVA and Tukey post-hoc tests, α = 0.05). *VuMGD1*, type 1 monogalactosyl-diacylglycerol synthase; *VuMGD2*, type 2 monogalactosyl-diacylglycerol synthase; *VuDGD1*, type 1 digalactosyl-diacylglycerol synthase; *VuDGD2*, type 2 digalactosyl-diacylglycerol synthase; *VuFAD7*, ω-3 fatty acid desaturase 7; *VuFAD8*, ω-3 fatty acid desaturase 8; *VuPAT1*, patatin-like lipid acyl hydrolase; *VuEF-1α,* elongation factor 1 alpha.

To examine further the interactive and individual effects of ozone and drought, we investigated the impact of the two stresses applied alone or in combination on pastidial galactolipids, which are known cellular targets of environmental stresses. We analyzed plastidial galactolipid content and fatty acid composition (Figure 2) as well as the transcript abundance of genes encoding enzymes involved in galactolipid synthesis, desaturation and degradation (Figure 3).

Galactolipids (monogalactosyl-diacylglycerol, MGDG and digalactosyl-diacylglycerol, DGDG) were the dominant lipid classes in cowpea leaf tissues, where their presence in plastidial membranes supports the bioenergetic and metabolic functions of the chloroplasts. The D treatment had no effect either on MGDG and DGDG contents (Figure 2a,b) or on their fatty acid composition and insaturation level (Table A1 and Table A3). In contrast, the O treatment affected plastidial galactolipids, in a significant manner in IT-83D (Figure 2a,b). In this cultivar, after 14 day of O treatment, MGDG and DGDG contents were reduced by 77% and 64%, respectively, as compared to the controls (Figure 2a,b), and these effects correlated with a 3-fold increase in the DGDG:MGDG ratio (Figure 2c). In the EPACE-1 cultivar, plastidial galactolipids were also affected by ozone (after 14 day MGDG and DGDG contents were reduced by 48% and 32%, respectively, as compared to the control), but these effects were not significant (Figure 2a,b) and the DGDG:MGDG ratio remained unchanged (Figure 2c). The polyunsaturated fatty acid 18:3 was predominant in the fatty acid composition of MGDG (ca. 95%) and DGDG (ca. 88%) in the leaves of both cultivars (Table A1). In EPACE-1, the FA composition of galactolipids was not significantly modified by the various treatments (Table A1 and Table A3). In IT83-D, the proportion of 18:3 in MGDG decreased from 95% in the control to 81% after 14 day of ozone treatment alone. This ozone-induced decrease in 18:3% was balanced by increases in the other FA % in MGDG, mainly the saturated FA 16:0 and 18:0 (Table A1) and translated into a decrease in the omega-3 index (Figure 2d). No apparent modification in FA % was observed in DGDG (Table A1).

### 2.3. Expression of Genes Coding Enzymes Involved in Lipid Metabolism

To better understand the observed modifications in plastidial galactolipid, the abundance of transcripts encoding enzymes involved in their biosynthesis, desaturation and degradation were analyzed using quantitative real-time PCR (Figure 3). The genes studied encoded two enzymes of the galactolipid biosynthesis pathway (MGDG synthase, EC 2.4.1.46: *VuMGD1* and *VuMGD2*; DGDG synthase, EC 2.4.1.241: *VuDGD1* and *VuDGD2*), two chloroplastic *ω*-3 fatty acid desaturases (EC 1.14.19.3, *VuFAD7* and *VuFAD8*) and the patatin-like lipid acyl hydrolase (EC 3.1.1.26: *VuPAT1*). Values obtained for the target genes were normalized with respect to the expression of the reference gene *VuEF1-α*.

In the control leaves of both cultivars, the expression level of most genes was higher at 14 day than at 7 day, by a factor of 2 to 10 (Figure 3). The O treatment had limited effects on the expression of the tested genes, except a down regulation of *VuMGD1* in EPACE-1 and *VuDGD1* in IT83-D at 7 day (Figure 3a,c), and a 27-fold up-regulation of *VuDGD2* in IT83-D at 14 day (Figure 3d). In contrast, the genes tested were globally down regulated in response to the D treatment, although a relatively high variability in the data limited the significance of the observed effects (Figure 3). Among the genes tested, *VuMGD2*, *VuDGD1* and *VuFAD8* showed the earliest and strongest repression under the drought treatment. As seen with most parameters that were analyzed in this study, the effects on transcipt levels of the analyzed genes of the OD treatment were essentially the same as those of the D treatment. In both cultivars, the expression of *VuPAT1* was unchanged, or slightly repressed, in response to the various treatments as compared to the control (Figure 3).

## 3. Discussion

### 3.1. The Drought Treament Restricted Plant Biomass Production but Did Not Cause Cellular Damage

The drought treatment caused an early and progressive stomatal closure in both cowpea cultivars (Figure 1b). As described in [30], the two cultivars showed a drought avoidance strategy through the early regulation of stomatal aperture, which allowed them to limit water loss as shown by the moderate reductions in RWC (Figure 1c). This, in turn, most likely restricted CO_2_ assimilation and explain the dramatic reduction in shoot biomass production after the first week of treatment (Figure 1a). Despite this strong restriction on plant growth and although the water-deprived plants showed signs of wilting, the results obtained on the first mature trifoliate leaves indicate that both cultivars were able to maintain cellular integrity under drought stress. First, leaves of drought-stressed plants did not display visible symptoms of injury or H_2_O_2_ accumulation (Figure A1); Second, membrane lipid content and composition were not modified in leaf tissues of plants submitted to the drought treatment alone (Figure 2 and Figure 3; Table A1 and Table A3). Contrary to the results of previous studies [29,30,38], IT83-D did not appear more sensitive to water stress than EPACE-1. It is likely that, in our experimental conditions, the severity of the drought treatment was not sufficient to reveal differences in drought tolerance between the two cultivars. Indeed, when compared to previous data obtained with the same cowpea cultivars submitted to drought, the RWC of 60%–70% measured at 14 day corresponds to a leaf water potential of −1.5 MPa [29], which is equivalent to a water stress defined as moderate in [31,39]. Consistent with this, several features of the responses of cowpea to severe drought, such as the decrease in leaf membrane lipid content [21,31] and the induction of *VuPAT1* expression [26], were not observed in the present work. Both cultivars maintained the FA unsaturation level of membrane lipids under drought stress (Table A1 and Table A3), even though *VuFAD7* and *VuFAD8* were repressed (Figure 3), which might indicate a capacity for stress acclimation [20].

### 3.2. The Ozone Treatment Caused Leaf Injury and Decreased Plastidial Galactolipid Content but Had Limited Effect at the Whole-Plant Level

In contrast with drought, the 14-day ozone treatment had a limited impact on photosynthesis and biomass production. These results are consistent with references [34,35]), where these parameters were strongly reduced but only after 40–50 days of treatment. Umponstira et al. [36] found strong ozone effects on cowpea biomass production after only 7 days of treatment, but subsequent results showed that cowpea plants at the vegetative growth stage, as in the present study, were less sensitive to ozone than plants at later growth stages [37]. In the present paper, significant effects of ozone were observed at the cellular level. Some of these effects were found in both cowpea cultivars. First, ozone exposure provoked symptoms of leaf injury and H_2_O_2_ accumulation (Figure A1). Ozone-induced cell death has been reported in various plant species and is associated to ROS accumulation [40]; Second, the ozone treatment induced decreases in the plastidial galactolipids MGDG and DGDG in both cultivars (Figure 2a,b). Reductions in galactolipid contents have also been reported in leaves of spinach [41] and snapbean [42] plants exposed to acute ozone exposure, and in pea and wheat plants subjected to moderate ozone concentrations [22,43]. Studies on the effects of short-term, acute exposure showed that the decrease in plastidial galactolipids did not result from the direct oxidative damage of ozone but rather from secondary effects on lipid metabolism [44,45]. The putative pathways for the degradation of galactolipids in ozone-treated leaves involves a number of enzymes, including one or several deacylating enzymes capable of hydrolyzing galactolipids, presumably lipid acyl hydrolases (LAHs) [46]. The expression of *VuPAT1*, a drought-inducible LAH purified from cowpea leaves with high substrate specificity for galactolipids [26], was very low in response to ozone in the present study, suggesting that other deacylating enzyme(s) are involved in galactolipid degradation in ozone-treated cowpea leaves.

### 3.3. Inter-Varietal Differences in Ozone Sensitivity

Most of the observed effects of ozone on cowpea leaves were more pronounced in IT83-D than in EPACE-1, including the extent of leaf injury and the decrease in galactolipid content. Intervarietal differences in ozone sensitivty were found in African cowpea cultivars, but could not be explained by differential radical scavenging capacities [34,35]. Here, this discrepancy between the two cultivars could be explained by a higher oxidative load in leaf tissues of IT83-D than in those of EPACE-1 as indicated by a higher ozone uptake in IT83-D (+21% in POD_0_, Table 1), even though this difference was not significant. Nevertheless, IT83-D showed responses to ozone that were not found in EPACE-1, such as the increase in the DGDG:MGDG ratio, as observed in snapbean cultivars subjected to acute ozone exposure [45,42]. The relative larger decline in MGDG is consistent with its extreme sensitivity to degradation processes activated by environmental stresses, as reported in drought-treated *Arabidopsis* [47] and cowpea plants [31]. Furthermore, the ozone treatment reduced the 18:3 in MGDG in IT83-D (Table A1), as found in the leaves of pea [22] and wheat [43] plants exposed to ozone. A LAH purified from cowpea leaves had the highest substrate specificity for (18:3/18:3)-MGDG [48] and LAH activities in cowpea leaf extracts were shown to selectively reduce 18:3 content in MGDG [49]. Taken together, these results suggest that the reduction in 18:3 content in MGDG reflects the preferential degradation of MGDG by LAHs upon ozone exposure in the sensitive cowpea cultivar IT83-D. Constraints such as wounding, low temperature and drought have been shown to increase the expression of the plastidial ω-3 fatty acid desaturase *FAD8* [39,50]. Furthermore, the over expression of *FAD8* increased 18:3 content and tolerance to salt and drought stresses in transgenic tobacco plants [51]. In the present work, the stimulation of *VuFAD8* expression found in IT83-D in response to ozone exposure might have mitigated the ozone-induced loss of 18:3 in membrane lipids but was not sufficient to maintain the unsaturation level of MGDG (Table A2). Beside their role in adjusting FA unsaturation, ω-3 FADs are involved in the regulation of plant defense responses through the production of trienoic FAs that serve as precursors for the synthesis of oxylipins such as jasmonic acid (JA) [52]. JA accumulates upon ozone exposure and acts in lesion containment during the process of ozone-induced cell death [17]. Since *VuFAD7* was repressed by the ozone treatment (Figure 3d), *VuFAD8* could have a specific role in the JA-mediated induction of defense responses to ozone in the leaves of the sensitive cowpea cultivar. Iyer et al. [10] showed that JA signaling was involved in the responses of *Medicago truncatula* to combined ozone and water stresses specifically. The potential role of *VuFAD8* in drought-ozone interactions could be further investigated with a longer water deprivation treatment.

Although the omega-3 index was determined from fatty acid contents in total membrane lipids, the lower 18:3 content in MGDG in IT83-D in response to ozone translated into a significant decrease of this biomarker. This indicates that the omega-3 index, which has been validated as an indicator of metal bioavailability in polluted soils and of the associated adverse effects on plants [53], can be used also as a relevant biomarker of ozone impact. However, the fact that the omega-3 index remained unchanged despite substantial ozone exposures in EPACE-1 in the O and OD treatments and in IT83-D in the OD treatment suggests that the biomarker’s response is not linearly related to the ozone dose and shows intraspecific variations.

### 3.4. Drought Alleviated the Effects of Ozone by Reducing Its Stomatal Uptake

Ozone is known to alter stomatal responses to a variety of environmental stimuli [54,55]. By inducing stomatal sluggishness [13] or by disrupting the ABA-induced signal transduction pathway for stomatal control [14], ozone can impair the ability of plants to respond to water stress. However, in our study, the rate and intensity of the stomatal closure caused by the water deficit was not modified by ozone in the OD treatment as compared to the D treatment (Figure 1b). Reciprocally, however, the stomatal closure induced by the water deficit decreased the uptake of ozone in EPACE-1 and IT83-D when drought and ozone were applied in combination (Table 1). Accordingly, several effects of ozone observed in the O treatment were less pronounced in the OD treatment, including leaf injury, H_2_O_2_ accumulation and galactolipid depletion (Figure A1 and Figure 2a,b). Furthermore, some effects of ozone in IT83-D were not found in the combined OD treatment, such as the decrease in the omega-3 index, the increase in DGDG:MGDG ratio and the coordinated up regulation of *VuDGD2* and *VuFAD8* (Figure 2c and Figure 3d). Taken together, these data suggest that the drought-induced stomatal closure mitigated the detrimental impacts of ozone on cowpea leaf tissues, which is consistent with the findings of meta-analyses of the effects of ozone on trees [56] and crops [9].

## 4. Materials and Methods

### 4.1. Plant Materials and Growth Conditions

Two cultivars of *Vigna unguiculata* (L.) Walp. were used in these experiments: EPACE-1 originating from the semi-arid Northeastern part of Brazil and IT83-D from humid regions in Southern China. Based on electrolyte leakage tests, these cultivars have been classified as drought-tolerant and drought-susceptible, respectively [38]. Seeds were germinated in 5-L pots (one seed per pot) containing a mixture of 0.4 kg of compost (N/P/K 14/16/18, 1.2 kg·m^−3^, Gramoflor Repiquage, France) and 15 g of fertilizing granules (Nutricote T-100, N/P/K/MgO 13/13/13/2; Fertil, Boulogne-Billancourt, France), under controlled conditions (photosynthetic photon flux density of 250 µmol·m^−2^·s^−1^, 14 h daylight, 25 °C/20 °C day/night temperatures and 80% relative humidity) for two weeks. All 48 pots (24 pots per cultivar) were dispatched in eight identical phytotronic chambers (ground surface 1.44 m^2^, height: 2 m) where the seedlings were allowed to acclimate for one week before stress treatments were applied as described in the following paragraph. At the onset of the treatments, i.e., three weeks after sowing, plants had two mature trifoliate leaves.

### 4.2. Stress Treatments

For 14 day, three-week old cowpea seedlings were exposed to the following treatments: control (C), drought (D), ozone (O) and combined ozone and drought (O + D). Control plants were exposed to ambient air and received 400 mL of water daily. Drought was applied by withholding watering from the beginning of the acclimation period to the end of the experiment. Pots were weighed daily to make sure that water loss and soil drying progressed at the same rate for all droughted plants. At the end of the experiment (14 day), droughted plants had been submitted to a three-week water deprivation period and all showed signs of wilting. Ozone stress was imposed by exposing plants to 120 ± 10 ppb of ozone through 12-h daily fumigations (11 a.m. to 11 p.m.). Ozone was produced from pure O_2_ by two ozone generators (OZ500; Fischer, Bonn, Germany and CMG3-3; Innovatec II, Rheinbach, Germany) and continuously monitored by an ozone analyzer (O341M; Environment S.A., Paris, France). For molecular and biochemical assays, the first fully developed trifoliolate leaves were collected after 7 and 14 day of treatments, immediately frozen in liquid N_2_ and stored at −80 °C for further analysis.

### 4.3. Shoot Biomass Production, Relative Water Content, Chlorophyll Fluorescence and Stomatal Conductance Measurements

Shoots were collected and weighed after 7 and 14 day of treatments. Fresh tissues were then dehydrated at 60 °C during 48 h before determination of shoot dry weight (SDW). Relative water content (RWC) of leaf tissues was assessed by determining fresh weight (FW), turgid weight (TW) and dry weight (DW) of 2 cm diameter leaf discs according to the following formula [57]:

RWC = ((FW − DW)/(TW − DW)) × 100
(1)

In vivo chlorophyll fluorescence was measured with a Fluorescence Monitoring System (FMS 1, Hansatech). Leaves were dark-adapted for 30 min before the minimal fluorescence (*F*_0_) was recorded. A saturating flash (7200 µmol·m^−2^·s^−1^ for 1 s) was applied to obtain the maximal fluorescence of dark-adapted leaves (*F*_m_). Actinic light (400 µmol·m^−2^·s^−1^) was then turned on to drive photosynthesis. When the leaves reached steady-state conditions, the steady-state fluorescence (*F*_s_) was recorded. A saturating flash (7200 µmol·m^−2^·s^−1^ for 1 s) and a dark pulse were then applied to obtain the maximal (*F*_m_′) and minimal (*F*_0_′) fluorescence of light-adapted leaves, respectively. The photochemical efficiency of PSII (*F*_v_/*F*_m_) and the quantum yield of PSII (Φ_PSII_) were calculated as follows:

*F*_v_/*F*_m_ = (*F*_m_ − *F*_o_)/F_m_ (2)

Φ_PSII_ = (*F*_m_′ − *F*_0_′)/*F*_m_′ (3)

Stomatal conductance to water vapor (*g_s_*) was measured after 7 and 14 day of experiment with a SC-1 portable leaf porometer (Decagon Devices, Inc., Pullman, WA, USA). Measurements were performed inside the phytotronic chambers, before the beginning of the ozone fumigation period, on one of the leaflets of the first fully developed trifoliolate leaf.

### 4.4. Ozone Exposure and Dose Indices

The AOT40 (ozone concentration accumulated over a threshold concentration of 40 ppb, under a minimum irradiance of 50 W·m^−2^, in ppb·h) index is defined as the sum of the difference between the hourly mean ozone concentration at the top of the canopy and 40 ppb, for all daylight hours within a specified time period [58]. Instantaneous ozone uptake was calculated from the monitoring of ozone concentration and stomatal conductance to water vapor (*g*_s_) as described in [59]. The accumulated stomatal flux or Phytotoxic Ozone Dose (POD, in mmol·m^−2^) was determined by summing hourly ozone uptakes. Since no dose-response relationship is available for *Vigna* or related species, the POD was calculated without any threshold of instantaneous ozone flux [58]. Measurements of *g_s_* were performed at 1, 3, 5, 8, 10, 12, 14 days of experiment for the calculation of each hourly ozone uptake of the day. For days when *g_s_* was not measured, an average value was calculated from the values recorded the flanking days.

### 4.5. DAB Staining

Hydrogen peroxide (H_2_O_2_) accumulation was investigated using 3,3′-diaminobenzidine (DAB) staining. DAB solution (1 mg·mL^−1^) was vacuum-infiltrated into 1-cm diameter leaf discs and the disks were left to impregnate for 24 h in the dark. Leaf discs were then discolored in three successive 95% ethanol baths and stored in glycerol 30%. Pictures were taken with a stereoscopic microscope (Nikon SMZ1000) under 0.8× magnification, coupled with a digital camera (Nikon D70S).

### 4.6. Leaf Lipid Extraction and Separation

Leaf samples were ground in liquid nitrogen and approximately 1 g was boiled in distilled water for 2 min to stop lipolytic activities. Lipophilic compounds were extracted in a chloroform:methanol:water (2:1:1, *v*:*v*:*v*) mixture [60], dried under nitrogen stream and immediately resuspended in 1 mL of an ethanol:toluene mixture (1:4, *v*:*v*) for storage at 4 °C. All extraction solutions were supplemented with 0.01% (*v*:*v*) butylated hydroxytoluene (BHT) to prevent lipid oxidation. Total lipids were separated by thin layer chromatography (TLC) on silica gel plates (G60; Merck) with the solvent system developed by [61]. Bands corresponding to lipid classes were visualized with primuline (0.01% in 80% acetone, *m*:*v*) under UV light and scraped. Lipids were then saponified and the obtained fatty acids were methylated with boron trifluoride [62]. Fatty acid methyl esters (FAMEs) were quantified relative to heptadecanoic acid (17:0), which was added as an internal standard before methylation.

### 4.7. GC-MS Analysis

FAMEs were separated using a gas chromatograph (Clarus 680, Perkin Elmer, Waltham, MA, USA) fitted with a fused silica capillary column (60 m × 0.25 mm i.d., 0.25 µm film thickness, Elite-WAX ETR, Perkin Elmer). Samples and standards were introduced by a 1 µL splitless injection system at 250 °C. Helium was used as the carrier gas at a constant flow of 1 mL·min^−1^. The oven temperature was programmed to rise continuously from 75 °C to 200 °C for a total run time of 60 min. Separated FAMEs were analyzed through electronic ionization (70 eV) with a single quadripole mass spectrometer (Clarus 600, Perkin Elmer). FAMEs were identified by comparing the obtained spectral data to a NIST database (National Institute of Standards Technology, Gaithersburg, MD, USA). Calibration standards were prepared from a commercially available mixture of standards (F.A.M.E. Mix, C8-C22 unsaturates, Supelco, Bellefonte, PA, USA), using heptadecanoic acid (17:0) as an internal standard. Calibration standards were analyzed in triplicate and linear standard curves were established for the methyl esters of the five main fatty acids found in membrane lipids of *Vigna* leaves (16:0, 18:0, 18:1; 18:2, 18:3) [21]. To maximize sensitivity and specificity, FAMEs were quantified in single-ion-recording (SIR) mode. Mass spectra of eluting FAME compounds were identified using the commercial mass spectral library supplied with the manufacturer’s software (TurboMass, Perkin Elmer). For each sample of total lipids, the responses (peak areas) of FAMEs were normalized separately to the response of the internal standard 17:0 and quantified using the corresponding calibration relationship. To determine the lipid content in a given leaf, the amounts of fatty acids were summed and the result expressed in relation to the leaf tissue dry weight. The omega-3 index as defined as the 18:3/(18:0 + 18:1 + 18:2) fatty acid ratio was determined as described in [53]. Omega-3 index values were normalized to the highest value measured at a given time point for a given cultivar.

### 4.8. RNA Extraction and cDNA Synthesis

Leaf samples were ground in liquid N_2_ with pestle and mortar and approximately 70 mg of powder were used for total RNA extraction, using the Qiagen RNeasy Plant Mini kit following the manufacturer’s instructions. Total RNA (50 µg) was treated with TURBO DNase (Invitrogen, Carlsbad, CA, USA). RNA concentration was measured using a Nanodrop ND-1000 spectrophotometer (NanoDrop Technologies, Wilmington, DE, USA) and RNA integrity was controlled by electrophoresis on 1.5% agarose gels. cDNA synthesis was performed on 800 ng of total RNA extracts by reverse transcription with the SuperScript III RT kit (Invitrogen) according manufacturer’s instructions. cDNA samples were kept at −20 °C before analysis.

### 4.9. Real-Time PCR Analysis

Leaf cDNAs were used for the detection of transcript accumulation for the following genes involved in membrane lipid biosynthesis and degradation: *VuMGD1, VuMGD2, VuDGD1*, *VuDGD2*, *VuFAD7*, *VuFAD8*, *VuPAT1*. Additional information on real-time PCR analyses is given in Table A5. Real-time PCR reactions were performed using the Power SYBR Green PCR Master Mix kit (Applied Biosystems, Carlsbad, CA, USA) in a StepOne Plus termocycler (Applied Biosystems). The 20 μL reaction mixtures contained 1 μL of cDNA (from 4 ng of reverse-transcribed RNA), 5 pmol of both sense and antisense primers and 10 μL of 2× SYBR Green DNA Polymerase mix. All samples were amplified under the following conditions: one denaturing step at 95 °C for 10 min, 40 cycles: 95 °C for 15 s (denaturing), 62 °C for 30 s (annealing), 72 °C for 30 s (elongation). Efficiencies of real-time PCR reactions were determined using dilution series of calibrator cDNA samples. Reactions were run in two replicates and the relative gene expression levels were normalized to the expression levels of a selected reference gene (elongation factor 1 alpha *VuEF-1α*). The reference gene *VuEF1α* was selected among a number of potential housekeeping genes using the GeNorm method [63].

### 4.10. Statistical Analysis

As described in the appropriate figure captions, parameters were measured on 3 or 4 plants per treatment, considered as independent biological replicates. At each time point and for a given cultivar, data from the different treatments (control, C; drought, D; ozone, O; ozone + drought OD) were submitted to a one-way analysis of variance (ANOVA) followed by Tukey’s post-hoc tests (α = 0.05). Homogenous subsets were indicated by appropriate letters. In addition, the effects of experimental factors (time, cultivar, drought and ozone) and their interactions were assessed through the multivariate ANOVA of the whole data set and the between-subject effects are included as supplementary information (Supporting Information Table A2, Table A3 and Table A4. Statistical analysis were conducted using the R software [64].

## 5. Conclusions

Both cowpea cultivars showed acclimatory responses to drought such as stomatal closure that led to growth impairment, but the water stress was not severe enough to cause leaf and membrane injury nor to reveal differences in drought tolerance between IT83-D and EPACE-1 cultivars. In contrast, the ozone treatment had a limited impact at whole-plant level but provoked leaf injury and altered membrane lipids. These effects were more pronounced in IT83-D, revealing intervarietal differences in ozone tolerance. When stresses were combined, ozone did not modify the stomatal response to drought and the observed effects on whole-plant physiology were essentially the same as when drought was applied alone. Conversely, the drought-induced stomatal closure appeared to alleviate ozone effects through the reduction of ozone uptake. Although the impact of the combined stresses on yield could not be evaluated in this study, the interactive effects of combined ozone and drought may play a major role for cowpea and others crops exposed in the field to a combination of the two stresses, and should be taken into account in environmental risk assessment for vegetation.

## Figures and Tables

**Table 1:** Twelve-hour mean ozone concentration and indices of ozone exposure (AOT40, ozone concentrations accumulated over a threshold of 40 ppb) and stomatal uptake (POD_0_, phytotoxic ozone dose, expressed as the accumulated stomatal flux of ozone) to which cowpea plants were subjected in the following treatments: Control (C), Drought (D), Ozone (O), Ozone + Drought (OD). Mean ± SD are shown (*n* = 4). Ozone concentration and AOT40 were averaged from the values recorded in 4 identical chambers. Ozone stomatal uptake (POD_0_) was averaged from 4 biological replicates. Control (C) and drought-treated (D) plants were supplied with activated charcoal-filtered air and ozone concentration was close to zero (nd, not detected).

Cultivar	Treatment	12-h Mean (O_3_) (ppb)	AOT40 (ppm·h)	POD_0_ (mmol·m^−2^)
EPACE-1	C	nd	nd	nd
	D			nd
	O	130.4 ± 5.2	16.7 ± 1.0	3.8 ± 0.7
	OD			2.9 ± 0.5
IT83-D	C	nd	nd	nd
	D			nd
	O	130.4 ± 5.2	16.7 ± 1.0	4.6 ± 1.2
	OD			3.1 ± 1.4

## Abbreviations

AOT40	ozone concentration accumulated over a threshold concentration of 40 ppb
DGDG	digalactosyl-diacylglycerol
*g*_s_	stomatal conductance
LAH	lipid acyl hydrolase
MGDG	monogalactosyl-diacylglycerol
POD	phytotoxic ozone dose
RWC	relative water content
*VuMGD*	monogalactosyl-diacylglycerol synthase
*VuDGD*	digalactosyl-diacylglycerol synthase
*VuFAD*	ω-3 fatty acid desaturase
*VuPAT1*	patatin-like lipid acyl hydrolase
Φ_PSII_	quantum yield of PSII

## Appendix A

Figure A1: Detection of hydrogen peroxide using DAB staining; Table A1: MGDG and DGDG fatty acid composition; Table A2: Analysis of variance (ANOVA) of physiological parameters; Table A3: Analysis of variance (ANOVA) of lipid contents; Table A4: Analysis of variance (ANOVA) of gene expression data; Table A5: Description of genes analyzed by RT-qPCR.

**Table A1:** (**a**) MGDG and (**b**) DGDG fatty acid composition in the leaves of *Vigna unguiculata* plants subjected to the following treatments: Control (C), Drought (D), Ozone (O), Ozone + Drought (OD). Values are expressed as percentages of the total fatty acid content in a given lipid class, averaged from three independent biological replicates. Data in bold belong to subsets that only partially merge the core data set, data in bold on a grey background belong to strictly separate subsets (one-way ANOVA and Tukey post-hoc tests, *α* = 0.05). MGDG, monogalactosyl-diacylglycerol; DGDG, digalactosyl-diacylglycerol; 16:0, hexadecanoic acid; 18:0, octadecanoic acid; 18:1, 9-octadecenoic acid; 18:2, 9,12-octadecadienoic acid; 18:3, 9,12,15-octadecatrienoic acid.

**(a) MGDG**							
**Cultivar**	**Time (d)**	**Treatment**	**%16:0**	**%18:0**	**%18:1**	**%18:2**	**%18:3**
EPACE-1	7	C	1.5	0.8	0.1	1.2	96.3
		D	1.4	1.0	0.1	1.2	96.3
		O	1.9	1.6	0.2	1.2	95.2
		OD	1.5	1.2	0.2	1.2	95.8
	14	C	1.6	0.8	0.2	1.0	96.4
		D	1.3	1.2	0.2	1.4	95.9
		O	2.3	1.7	0.3	1.5	94.2
		OD	1.2	1.2	0.3	1.4	96.0
IT83-D	7	C	1.6	1.3	0.2	1.6	95.3
		D	1.7	1.2	0.2	1.4	95.5
		O	1.2	0.6	0.1	1.4	96.7
		OD	0.9	0.6	0.1	1.3	97.0
	14	C	1.9	1.2	0.3	1.2	95.5
		D	1.7	2.0	0.3	1.6	94.5
		O	6.4	7.7	1.1	3.1	81.6
		OD	2.1	2.4	0.4	1.8	93.4
							
**(b) DGDG**							
**Cultivar**	**Time (d)**	**Treatment**	**%16:0**	**%18:0**	**%18:1**	**%18:2**	**%18:3**
EPACE-1	7	C	7.3	1.7	0.2	1.0	89.8
		D	8.1	2.4	0.3	1.1	88.1
		O	7.7	2.9	0.4	1.1	87.9
		OD	8.8	2.8	0.3	1.2	86.9
	14	C	7.7	2.2	0.3	1.1	88.7
		D	6.9	2.8	0.3	1.3	88.7
		O	8.1	2.7	0.5	1.2	87.6
		OD	10.5	4.1	0.4	1.4	83.7
IT83-D	7	C	8.6	2.1	0.3	1.2	87.8
		D	3.7	1.9	0.7	2.1	91.7
		O	8.0	1.5	0.2	1.0	89.3
		OD	6.8	1.6	0.4	1.1	90.1
	14	C	8.1	4.0	0.5	1.5	85.9
		D	6.5	3.5	0.4	1.4	88.2
		O	7.2	6.3	1.0	2.0	83.5
		OD	7.8	4.7	0.6	1.6	85.2

**Table A2:** Analysis of variance (ANOVA) of physiological parameters. The displayed results are *p*-values for individual factors (time, cultivar, drought and ozone) and their multiple interactions. Values in bold are significant at α = 0.05. SDW, shoot dry weight; *g*_s_, stomatal conductance; RWC, relative water content; Φ_PSII_, actual quantum yield of PSII; *F*_v_/*F*_m_, maximum quantum yield of PSII.

Factor	SDW	*g*_s_	RWC	Φ_PSII_	*F*_v_/*F*_m_
Time (*t*)	0.000	0.004	0.000	0.000	0.000
Cultivar (cv)	0.743	0.609	0.459	0.000	0.000
Drought (D)	0.000	0.000	0.000	0.000	0.004
Ozone (O)	0.194	0.000	0.817	0.141	0.656
t × cv	0.960	0.594	0.055	0.094	0.923
t × D	0.000	0.477	0.000	0.103	0.096
cv × D	0.809	0.341	0.012	0.091	0.813
t × cv × D	0.415	0.468	0.420	0.091	0.713
t × O	0.777	0.811	0.040	0.494	0.002
cv × O	0.303	0.595	0.726	0.122	0.035
t × cv × O	0.881	0.300	0.126	0.063	0.022
D × O	0.084	0.000	0.844	0.242	0.736
t × D × O	0.677	0.770	0.895	0.763	0.213
cv × D × O	0.387	0.977	0.890	0.590	0.559
t × cv × D × O	0.442	0.866	0.037	0.850	0.253

**Table A3:** Analysis of variance (ANOVA) of lipid contents. The displayed results are *p*-values for individual factors (time, cultivar, drought and ozone) and their multiple interactions. Values in bold are significant at α = 0.05. MGDG, monogalactosyl-diacylglycerol; DGDG, digalactosyl-diacylglycerol.

Factor	MGDG	DGDG
Time (*t*)	0.000	0.003
Cultivar (cv)	0.961	0.180
Drought (D)	0.987	0.679
Ozone (O)	0.002	0.000
t × cv	0.131	0.466
t × D	0.977	0.804
cv × D	0.514	0.318
t × cv × D	0.819	0.499
t × O	0.094	0.396
cv × O	0.150	0.026
t × cv × O	0.269	0.875
D × O	0.464	0.798
t × D × O	0.696	0.660
cv × D × O	0.279	0.264
t × cv × D × O	0.734	0.899

**Table A4:** Analysis of variance (ANOVA) of gene expression data. The displayed results are *p*-values for individual factors (time, cultivar, drought and ozone) and their multiple interactions. Values in bold are significant at α = 0.05. *VuMGD1*, type 1 monogalactosyl-diacylglycerol synthase; *VuMGD2*, type 2 monogalactosyl-diacylglycerol synthase; *VuDGD1*, type 1 digalactosyl-diacylglycerol synthase; *VuDGD2*, type 2 digalactosyl-diacylglycerol synthase; *VuFAD7*, ω-3 fatty acid desaturase 7; *VuFAD8*, ω-3 fatty acid desaturase 8; *VuPAT1*, patatin-like lipid acyl hydrolase.

Factor	*VuMGD1*	*VuMGD2*	*VuDGD1*	*VuDGD2*	*VuFAD7*	*VuFAD8*	*VuPAT1*
Time (*t*)	0.000	0.033	0.000	0.000	0.000	0.000	0.729
Cultivar (cv)	0.000	0.348	0.066	0.000	0.789	0.000	0.414
Drought (D)	0.000	0.005	0.000	0.000	0.000	0.000	0.010
Ozone (O)	0.110	0.102	0.986	0.000	0.525	0.000	0.026
t × cv	0.000	0.310	0.131	0.000	0.592	0.000	0.559
t × D	0.000	0.034	0.000	0.000	0.000	0.000	0.026
cv × D	0.000	0.271	0.058	0.000	0.948	0.000	0.208
t × cv × D	0.000	0.251	0.110	0.000	0.994	0.000	0.578
t × O	0.016	0.151	0.532	0.000	0.600	0.000	0.067
cv × O	0.000	0.614	0.000	0.000	0.885	0.000	0.064
t × cv × O	0.000	0.629	0.000	0.000	0.687	0.000	0.147
D × O	0.037	0.106	0.840	0.000	0.380	0.000	0.028
t × D × O	0.001	0.141	0.559	0.000	0.591	0.000	0.035
cv × D × O	0.000	0.623	0.000	0.000	0.205	0.000	0.232
t × cv × D × O	0.000	0.632	0.000	0.000	0.115	0.000	0.221

**Table A5:** Name, function and accession number of genes measured by real-time quantitative PCR as well as sequences of sense and antisense primers used for qPCR analyses.

Gene Name	Gene Function	GenBank Accession Number
*VuMGD1*	Type 1 monogalactosyl-diacylglycerol synthase	DQ205521
*VuMGD2*	Type 2 monogalactosyl-diacylglycerol synthase	EF466098
*VuDGD1*	Type 1 digalactosyl-diacylglycerol synthase	DQ205523
*VuDGD2*	Type 2 digalactosyl-diacylglycerol synthase	EF466099
*VuFAD7*	ω-3 fatty acid desaturase 7	EU180596
*VuFAD8*	ω-3 fatty acid desaturase 8	EU180595
*VuPAT1*	Patatin-like lipid acyl hydrolase	AF193067
*VuEF-1α*	Elongation factor 1 alpha	HO223992
**Gene Name**	**Sense Primer**	**Antisense Primer**
*VuMGD1*	5′GTCCATCCACTGATGCAGCAC3′	5′TTGCGCAACATCTGTTGTAGG3′
*VuMGD2*	5′GTCCATCCACTGATGCAGCAC3′	5′ATTGACCCTTCACAAGAACC3′
*VuDGD1*	5′GTAATTTGCAATGTTCATGGTGT3′	5′TCTGAACTTCATTAGCATCCTCTC3′
*VuDGD2*	5′TGCACAGCCTACTAATGCTGAG3′	5′TGCAAGGTATGTGGAATAGCAC3′
*VuFAD7*	5′GCTTCAATCTTGAGTCCTATGG3′	5′CCAACCTTGGAGGAGCTGGAC3′
*VuFAD8*	5′ACCAGTTCTTGGTCAATATTACCG3′	5′CAGTGACTTCTCTCAGTCTTC3′
*VuPAT1*	5′TTTGCTTGCTTTCCTCGAAT3′	5′CGGGAAGATTTTTGGGGTAT3′
*VuEF-1α*	5′GTAACAAGATGGATGCCACC3′	5′CCACTTTCTTCAAATACGAGGAG3′
